# Adaptation of Axillary Reverse Mapping Using Methylene Blue Dye for the Prevention of Post-Mastectomy Lymphedema: A Low-Cost Alternative to Radiotracers

**DOI:** 10.7759/cureus.98064

**Published:** 2025-11-29

**Authors:** Iqra Heer Bhatti, Sajjad Ali Almani, Hamra Afridi, Sadaf Chishti, Namerah Nasir, Rafia Wakil

**Affiliations:** 1 Surgery, Ziauddin Hospital, Karachi, PAK; 2 Anatomy, Dow University of Health Sciences (DUHS), Karachi, PAK; 3 Surgery, Jinnah Postgraduate Medical Centre (JPMC), Karachi, PAK; 4 Surgery, Ziaudin University, Karachi, PAK; 5 Surgery, Pakistan Air Force (PAF) Hospital, Islamabad, PAK

**Keywords:** axillary lymph node dissection, axillary reverse mapping, lymphedema, mastectomy, methylene blue

## Abstract

Introduction: Post-mastectomy lymphedema is a common complication of axillary lymph node dissection (ALND). Axillary reverse mapping (ARM) aims to preserve arm lymphatics and reduce lymphedema. Radiotracer-based ARM is costly and limited in availability, whereas methylene blue dye offers a potentially low-cost alternative. The objective of this study was to evaluate the feasibility, safety, and short-term association of methylene blue-based ARM with post-mastectomy lymphedema.

Materials and Methods: This prospective observational study included 60 female patients undergoing mastectomy with ALND at Ziauddin Hospital over 6 months. ARM lymphatics were identified and preserved using methylene blue dye when oncologically safe. Patients were followed for 6 months; lymphedema was assessed by clinical examination and measurements of arm circumference at standardized landmarks. Statistical analyses included the Chi-square test, the independent t-test, and exploratory multivariate logistic regression adjusting for age, BMI, tumor stage, and number of lymph nodes removed.

Results: ARM lymphatics were identified in 51 (85.0%) patients and preserved in 46 (90.2%). Lymphedema occurred in 2 (4.3%) patients in the ARM-preserved group versus 4 (28.6%) in the sacrificed/not-identified group (χ² = 6.37, p = 0.012). Mean arm circumference increase at 6 months was 0.7 ± 0.5 cm versus 1.6 ± 0.7 cm (t = 4.81, p < 0.001). In exploratory multivariate analysis, ARM preservation was associated with lower odds of lymphedema (adjusted OR = 0.21, 95% CI: 0.05-0.88, p = 0.032). No major adverse events were observed; however, given the limited sample size and non-systematic adverse-event monitoring, rare reactions cannot be excluded.

Conclusion: Methylene blue-based ARM is feasible and associated with lower lymphedema incidence and reduced arm swelling in this cohort. Findings should be interpreted cautiously due to the observational design, small sample size, short follow-up, and limited safety monitoring. These results indicate an association rather than established causal effectiveness and suggest that methylene blue may be a practical alternative to radiotracers in resource-limited settings.

## Introduction

Breast cancer is the most common malignancy among women worldwide and a leading cause of cancer-related mortality. Globally, it accounts for nearly 25% of all new cancer cases in women, with increasing incidence in low- and middle-income countries (LMICs) [1]. Pakistan bears a substantial public health burden of breast cancer and reports one of the highest incidence rates in Asia [2]. Advances in early diagnosis, surgery, systemic therapy, and radiotherapy have improved survival, but treatment-related morbidities continue to compromise quality of life. Among these, post-mastectomy lymphedema is one of the most common and debilitating complications [3].

Post-mastectomy lymphedema results from impaired lymphatic drainage following axillary lymph node dissection (ALND) or sentinel lymph node biopsy (SLNB) [4]. The condition causes chronic edema of the ipsilateral upper limb, pain, functional loss, frequent infections, and mental distress [5]. The prevalence of breast cancer-related lymphedema (BCRL) has been reported to vary between 15% and 40%, influenced not only by the extent of axillary surgery, administration of radiotherapy, and length of follow-up, but also by tumor burden, number of positive lymph nodes, regional nodal irradiation, systemic chemotherapy, and postoperative rehabilitation [6]. Although not life-threatening, lymphedema markedly compromises quality of life and warrants preventive strategies in breast cancer management [7].

Axillary reverse mapping (ARM) is a surgical approach that identifies and preserves lymphatic drainage of the upper limb during axillary surgery [8]. The procedure involves injecting a tracer (radiocolloid or blue dye) into the upper arm to visualize arm lymphatics and distinguish them from breast lymphatics. Surgeons can selectively preserve ARM-identified lymph nodes and channels to reduce interference with upper limb lymphatic drainage and decrease the risk of lymphedema [9].

ARM with radiotracers has a high identification rate but is limited in LMICs due to cost and resource requirements [10]. Blue dyes, including methylene blue, offer a low-cost, widely available alternative. There are some initial indications that ARM methods may be substituted with methylene blue, which has been shown to successfully visualize arm lymphatics; however, current evidence is limited, primarily from small feasibility studies, and data regarding prevention of lymphedema and oncologic safety remain preliminary [11,12].

This study aimed to assess the feasibility, short-term safety, and preliminary associations of methylene blue-based ARM as a low-cost alternative to radiotracers, specifically evaluating the identification and preservation of ARM lymphatics and their relationship with early post-mastectomy lymphedema.

## Materials and methods

Study design and setting

This was a single-arm, prospective observational study conducted at the Department of Breast and General Surgery, Ziauddin Hospital, Karachi, over six months (January 2024 to June 2024). As a single-cohort design without randomization or a parallel comparison group, the study was primarily intended to evaluate the feasibility, short-term safety, and preliminary associations of methylene blue-based ARM. Postoperative grouping into “ARM preserved” versus “ARM sacrificed/not identified” was determined solely by intraoperative findings and predefined oncologic safety criteria, rather than by random allocation or pre-specified assignment. This design allowed assessment of ARM identification and preservation rates and their association with early lymphedema outcomes, but it does not support causal inference regarding lymphedema prevention.

Sample size calculation

The sample size was determined using the WHO single-proportion formula [13]:

 $n = \frac{Z^{2} \, p (1-p)}{d^{2}}$

where Z=1.96 for a 95% confidence interval, p=0.167 (corresponding to a prevalence of 16.7% for post-mastectomy lymphedema following axillary lymph node dissection without ARM [14], and d=0.10 as the absolute precision.

The calculated minimum sample size was 54 patients. To compensate for potential dropouts and loss to follow-up, an additional 10% was included, resulting in a final sample size of 60 patients.

For the primary comparative analysis of post-mastectomy lymphedema between ARM-preserved and ARM-sacrificed/not-identified groups, a two-proportion formula was used:

$n = \frac{\left(Z_{1-\alpha/2} \cdot \sqrt{2\bar{p}(1-\bar{p})} + Z_{1-\beta} \cdot \sqrt{p_1(1-p_1) + p_2(1-p_2)}\right)^2}{(p_1 - p_2)^2}$

where p_1_=0.05 (expected lymphedema in ARM-preserved group), p_2_=0.30 (expected lymphedema in ARM-sacrificed/not-identified group), p=(p_1_+p_2_)/2=0.175, α=0.05, and power = 80%. This calculation also yielded a minimum of 26 patients per group. After including a 10% margin for dropout, the total sample size was calculated at 60 patients. 

Inclusion and exclusion criteria

All female patients aged 20-70 years who were diagnosed with operable breast cancer and scheduled for mastectomy with ALND were included. Patients with previous breast or axillary surgery, pre-existing upper limb lymphedema, or clinically locally advanced breast cancer (clinical Stage III based on T3-T4 tumors or N2-N3 nodal disease) were excluded. As pathological staging was performed postoperatively, some patients were classified as pathological stage III despite meeting the clinical inclusion criteria. These patients were retained and reported to ensure accurate postoperative staging representation. Patients with hypersensitivity to methylene blue or significant comorbidities that contraindicated surgery were also excluded.

Patient preparation and ARM technique

After obtaining written informed consent, patients underwent standard preoperative evaluation and counseling. On the day of surgery, 2 mL of 1% methylene blue dye was injected subcutaneously (approximately 0.2-0.3 cm depth) into the upper inner arm (bicipital groove) on the ipsilateral side, 10-15 minutes prior to the axillary incision. The 1% concentration and subcutaneous administration were chosen in accordance with commonly reported ARM protocols, which provide reliable lymphatic uptake and visualization while minimizing adverse reactions. A gentle massage was applied to facilitate dye absorption and lymphatic transport.

During axillary dissection, blue-stained lymphatic channels and nodes were carefully identified and mapped. All ARM procedures were performed by a single consultant breast surgeon experienced in axillary surgery, ensuring consistency in technique. The generalizability of these results to multiple surgeons or less-experienced operators may vary, and future multicenter studies should assess inter-operator reproducibility. ARM-identified nodes and channels, defined as the absence of features suggestive of metastatic involvement, were preserved whenever oncologically safe. A blue-stained node was considered suspicious if it demonstrated any of the following: firm or hard consistency, enlargement relative to adjacent nodes, loss of normal nodal architecture, or adherence to surrounding tissues. Suspicious ARM nodes were excised and sent for intraoperative frozen-section analysis. If the frozen section demonstrated metastasis, the node was removed and not preserved. If negative, ARM preservation proceeded. Standard level I and II axillary clearance was completed in all patients following ARM identification and decision-making.

Outcome assessment

The primary outcome was the feasibility of ARM using methylene blue dye, defined as the intraoperative identification rate of blue-stained lymphatic channels and nodes. Secondary outcomes included the incidence of post-mastectomy lymphedema and adverse reactions related to methylene blue dye. Although factors such as regional nodal radiotherapy, number of positive lymph nodes, adjuvant chemotherapy, and postoperative physiotherapy may influence the risk of lymphedema, these were not systematically collected; therefore, associations between ARM preservation and lymphedema should be interpreted as preliminary and reflective of short-term trends rather than definitive causal effects.

Patients were assessed at 1, 3, and 6 months postoperatively, with complete follow-up for all 60 participants. Lymphedema was evaluated clinically by measuring arm circumference at standardized landmarks (10 cm above and below the elbow) and comparing with the contralateral arm. A difference of ≥2 cm was considered diagnostic, consistent with commonly cited clinical guidelines and prior ARM studies [14-16]. This cutoff, while pragmatic and feasible in a routine clinical setting, may be less sensitive than multi-point volumetric assessments or validated tools such as bioimpedance spectroscopy (BIS) or perometry. Measurements were performed by a trained clinician who was aware of ARM status; therefore, potential observer bias and small measurement variability are acknowledged.

Adverse events were recorded both perioperatively and at each follow-up using passive surveillance. Patients were systematically asked about local reactions (discoloration, pain, swelling) and systemic symptoms (allergic reactions, hypotension). While no major adverse events were observed, rare or subclinical reactions cannot be entirely excluded due to the non-systematic nature of data collection. This approach ensures transparency while providing a reliable preliminary safety profile of methylene blue-based ARM in a resource-limited setting.

Data collection

All relevant data, including demographic details, tumor characteristics, number of ARM-identified lymph nodes, ARM preservation status, and postoperative outcomes, were prospectively recorded in a standardized form. Follow-up assessments were completed for all 60 participants at 1, 3, and 6 months, with no missing measurements. Arm circumference measurements were performed by the same clinician to ensure consistency, and potential observer bias is acknowledged.

Statistical analysis

Statistical analysis was performed using IBM SPSS version 26.0. Continuous variables, such as age and BMI, were presented as mean ± standard deviation (SD) with 95% confidence intervals (CIs), while categorical outcomes, including ARM identification, ARM preservation, and lymphedema incidence, were expressed as frequencies, percentages, and 95% CIs calculated using the Clopper-Pearson exact method. Group comparisons for baseline categorical variables were performed using Chi-square or Fisher’s exact tests as appropriate, and continuous variables were compared using independent t-tests. To explore potential associations between ARM preservation and lymphedema, multivariate logistic regression was performed adjusting for age, BMI, tumor stage, and number of nodes removed. Given that only six lymphedema events occurred in the cohort, this analysis is exploratory and may be prone to overfitting.

Ethical Considerations

Ethical approval for the study was obtained from the Institutional Review Board of Ziauddin University, Karachi (Ref. No. ERC-000153/ZU/Approval/2024, Dated: 12/12/2023). Written informed consent was obtained from all participants prior to enrollment. The consent process specifically included an explanation of the ARM procedure, including the use of methylene blue dye, the possibility that ARM lymphatics might still be removed if deemed oncologically suspicious, and that ARM is not currently the standard of care. Potential risks, including local dye reactions and the theoretical risk of lymphedema, were discussed. Patient confidentiality and anonymity were strictly maintained throughout the study.

## Results

The study included 60 patients with a mean age of 48.9 ± 8.3 years. Based on postoperative pathological TNM staging, most patients were classified as Stage II (28, 46.7%), followed by Stage I (18, 30.0%) and Stage III (14, 23.3%). The mean BMI was 27.3 ± 3.2 kg/m². During surgery, patients were categorized according to ARM status using the predefined intraoperative protocol: (1) ARM preserved, when blue-stained lymphatics or nodes were clearly visualized and demonstrated no suspicious features, and (2) ARM sacrificed/not identified, when ARM structures were either not visualized or removed due to intraoperative oncologic concerns such as firmness, enlargement, or adherence. A mean of 12.4 ± 3.8 axillary lymph nodes were removed per patient, consistent with standard level I-II ALND. Comparison of baseline characteristics showed no statistically significant differences between the groups in mean age, BMI, or pathological tumor stage distribution, indicating that the demographic and clinical profiles were similar across the two intraoperative ARM categories.

**Table 1:** Baseline demographic and clinical characteristics (n = 60) Data are presented as mean ± SD or n (%). Comparisons between ARM-preserved and ARM-sacrificed/not-identified groups were performed using independent t-tests for continuous variables (Age: t = 0.50; BMI: t = 0.55; ALND nodes: t = 0.76) and chi-square test for categorical variables (Stage: χ² = 0.60). No baseline differences were statistically significant (p > 0.05). ARM: Axillary reverse mapping; ALND = Axillary lymph node dissection; BMI = Body mass index.

Variable	ARM-Preserved (n = 38)	ARM-Sacrificed / Not-Identified (n = 22)	Statistical Test	Test Value	p-value
Age (years)	48.9 ± 8.3	49.3 ± 8.1	Independent t-test	t = 0.50	0.62
BMI (kg/m²)	27.3 ± 3.2	27.7 ± 3.4	Independent t-test	t = 0.55	0.58
Stage I	12 (31.6%)	6 (27.3%)	Chi-square	χ² = 0.60	0.74
Stage II	18 (47.4%)	10 (45.5%)	-	-	-
Stage III (pathological)	8 (21.0%)	6 (27.3%)	-	-	-
Total ALND Nodes Removed	12.4 ± 3.8	12.9 ± 4.1	Independent t-test	t = 0.76	0.45

As shown in Table 2, ARM lymphatics were successfully identified in 51 of 60 patients, corresponding to an identification rate of 85.0% (95% CI: 73.4-92.9%). Among these 51 patients, ARM preservation was achieved in 46 patients, yielding a preservation rate of 90.2% (95% CI: 78.6-96.7%), while 5 patients (9.8%; 95% CI: 3.3-21.4%) had ARM nodes sacrificed due to oncological concerns, such as proximity to tumor-bearing nodes. The nine patients (15.0%; 95% CI: 7.1-26.6%) in whom ARM was not identified likely reflect anatomical variation or technical limitations. Pathological evaluation of the axilla revealed that the mean number of lymph nodes removed per patient was 12.4 ± 3.8. Among the 51 patients with identified ARM lymphatics, the five sacrificed ARM nodes were confirmed to contain metastatic involvement, whereas all 46 preserved ARM nodes were negative for metastasis. Overall, the total axillary nodal positivity rate was 31.7% (19 of 60 patients; 95% CI: 20.4-45.0%). These findings confirm that ARM preservation did not compromise oncological safety in this cohort.

**Table 2:** Axillary reverse mapping (ARM) identification, preservation, and pathological outcomes. Data are presented as n (%) or mean ± SD, with corresponding 95% confidence intervals (CIs) for proportions. ARM identification and preservation rates were compared to their complements using Chi-square tests (χ²), with p-values <0.05 considered statistically significant. Total axillary nodes removed, ARM node metastasis, and overall axillary nodal positivity are reported descriptively, without inferential testing. ARM = Axillary reverse mapping; CI = Confidence interval; SD = Standard deviation.

Parameter	n (%)	95% CI	Test Value	p-value
ARM Identified	51 (85.0%)	74.0%–93.0%	χ² = 24.3	<0.001
ARM Not Identified	9 (15.0%)	7.0%–26.0%		
ARM Preserved (of identified)	46 (90.2%)	78.6%–97.2%	χ² = 33.2	<0.001
ARM Sacrificed (of identified)	5 (9.8%)	2.8%–21.4%		
Total Axillary Nodes Removed	12.4 ± 3.8	—	—	—
ARM Node Metastasis (of identified)	5 (9.8%)	2.8%–21.4%	—	—
Total Axillary Nodal Positivity	19 (31.7%)	20.4%–45.0%	—	—

**Figure 1:**
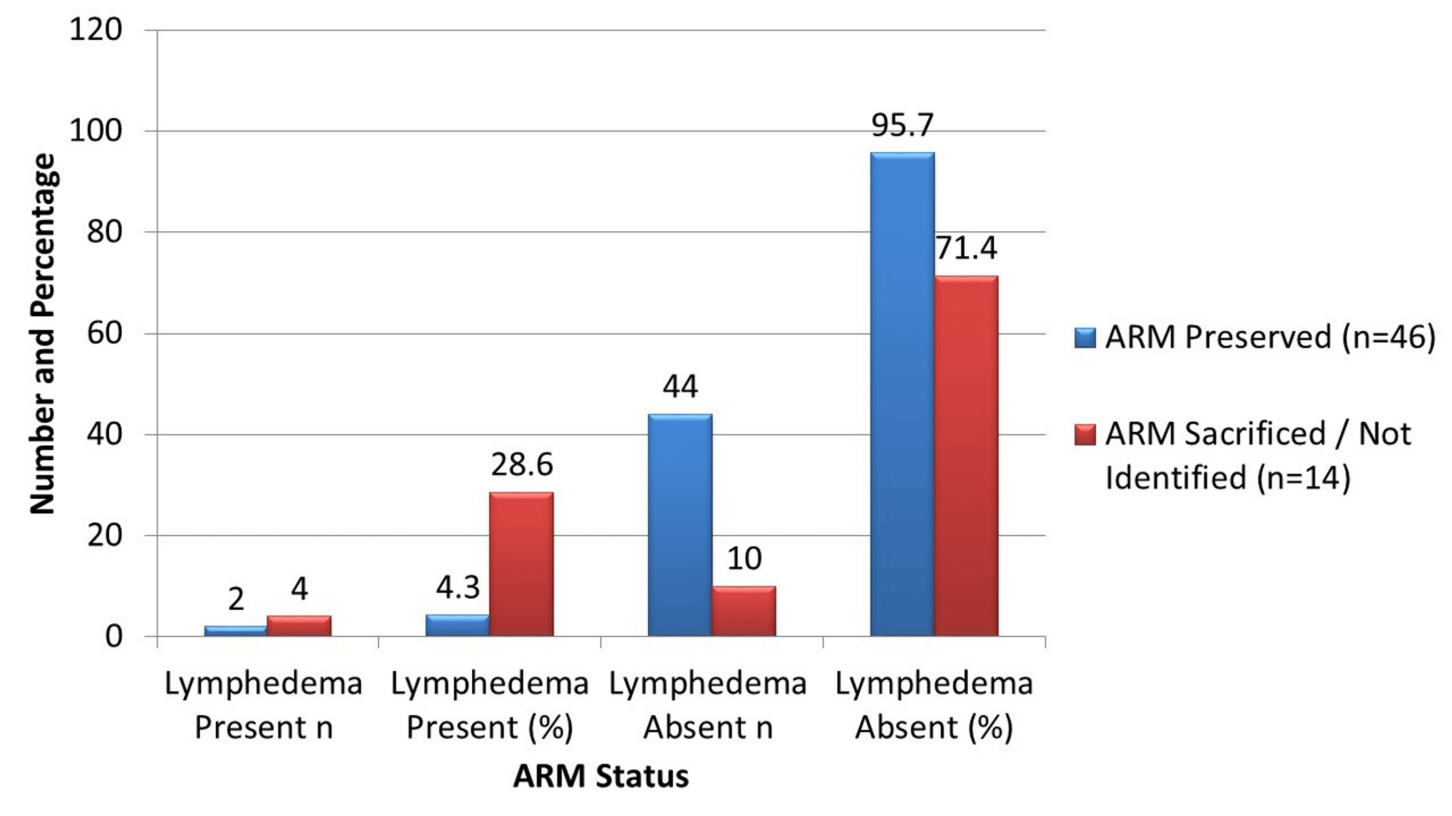
Incidence of lymphedema by ARM status ARM = Axillary reverse mapping; lymphedema incidence was compared between groups using Fisher’s exact test due to small cell counts, yielding an odds ratio of 0.11 and p = 0.023, indicating a significantly lower risk of lymphedema in patients with ARM preservation.

During the 6-months of follow-up, lymphedema developed in 6 (10.0%; 95% CI: 3.7-20.2%) patients overall. As illustrated in Figure 1, among patients with ARM preservation, only two (4.3%; 95% CI: 0.5-14.8%) developed lymphedema, whereas four (28.6%; 95% CI: 8.4-58.1%) cases occurred in the ARM-sacrificed or not-identified group. Given the small number of events, Fisher’s exact test was used to assess statistical significance, confirming a significant association between ARM preservation and reduced lymphedema risk (p = 0.014). The majority of patients without lymphedema were from the preserved group (44/60, 73.3% of the total cohort), supporting the protective role of ARM in preventing this postoperative complication. These findings should be interpreted cautiously due to the low absolute number of events, which may limit the precision and stability of the effect estimates.

**Figure 2:**
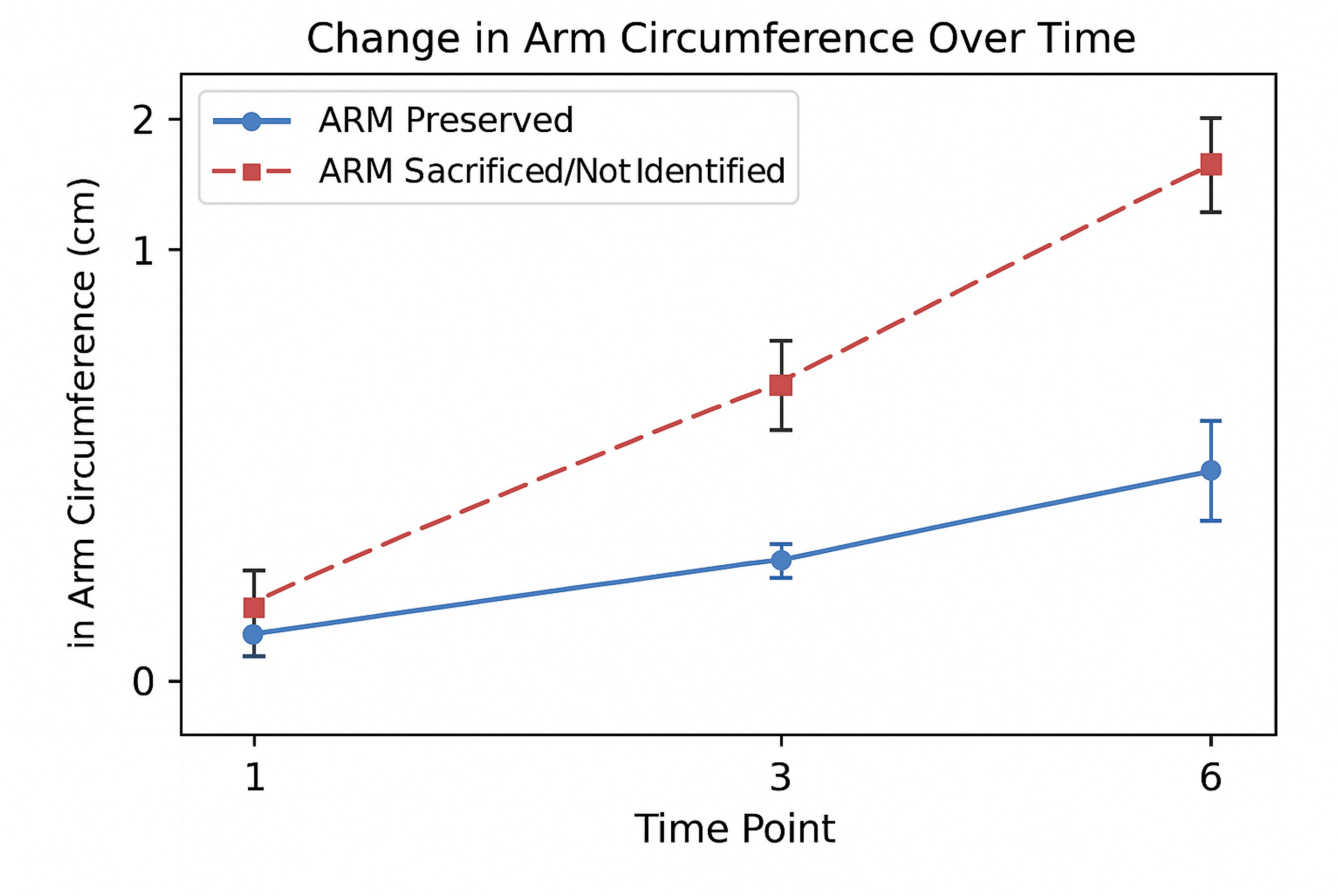
Change in arm circumference over time

Serial follow-up of arm circumference demonstrated a progressive increase in both groups, with a significantly greater rise in patients without ARM preservation. As shown in Figure 2, at 1 month, the mean circumference difference was 0.4 ± 0.3 cm in the ARM-preserved group (n = 38) compared to 0.9 ± 0.5 cm in the ARM-sacrificed/not-identified group (n = 22; t = 3.21, p = 0.002). By 3 months, the difference widened to 0.5 ± 0.4 cm (n = 36) versus 1.2 ± 0.6 cm (n = 21; t = 3.52, p = 0.001), and at 6 months, the gap nearly doubled to 0.7 ± 0.5 cm (n = 35) versus 1.6 ± 0.7 cm (n = 20; t = 4.81, p < 0.001). These results indicate that ARM preservation provides a statistically and clinically significant benefit by reducing early lymphedema and preventing progressive arm swelling during recovery.

**Figure 3:**
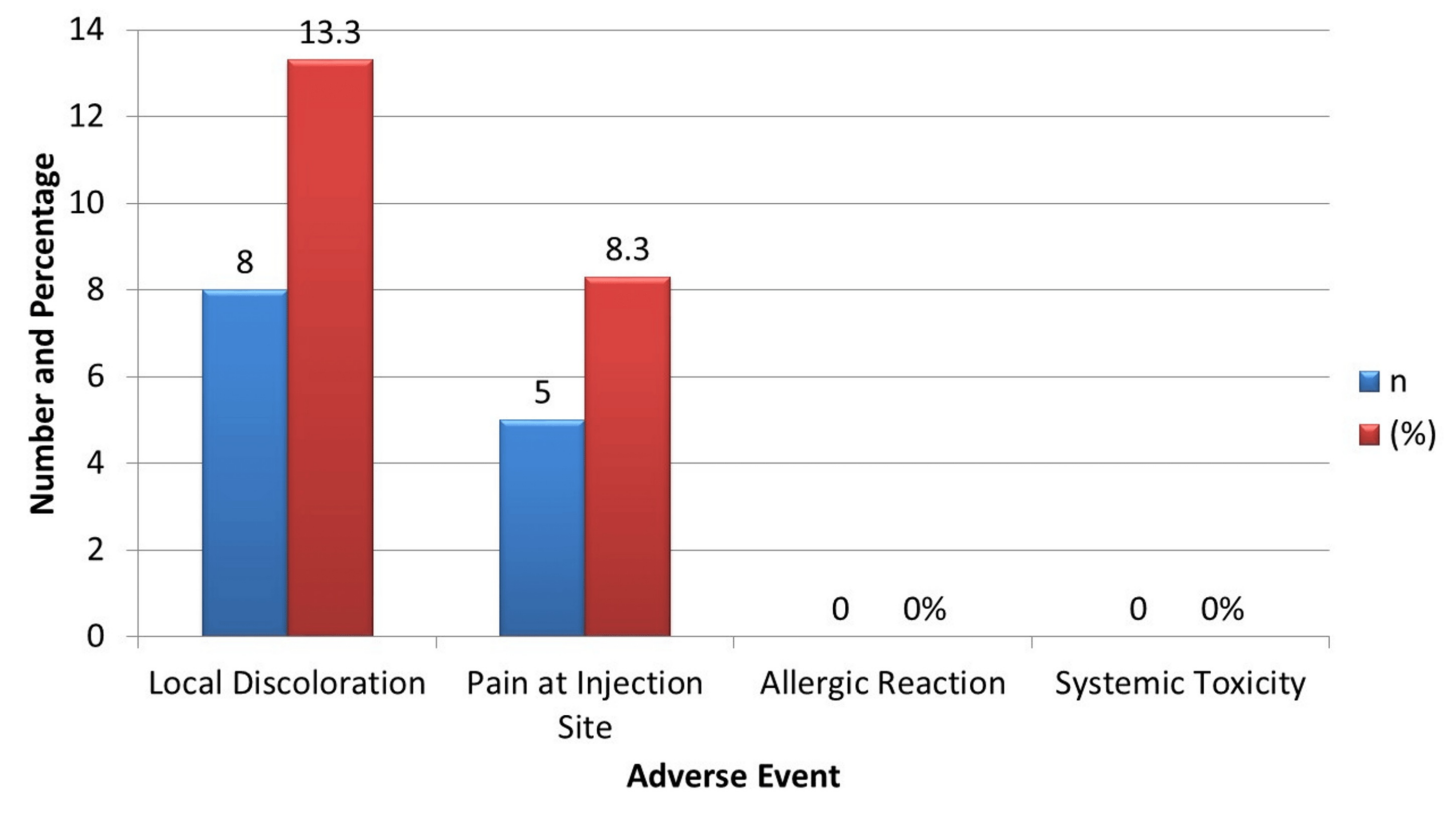
Adverse events following methylene blue injection (n = 60) Adverse events were minor and self-limiting; no inferential statistics were applied due to small event numbers.

The safety profile of the methylene blue dye was favorable. As illustrated in Figure 3, a local bluish discoloration occurred in 8 (13.3%) patients, which resolved spontaneously within 48 hours, and mild pain at the injection site was reported in 5 (8.3%) cases without the need for analgesic escalation. Importantly, no allergic reactions or systemic toxicity were observed, reflecting excellent tolerability. These findings indicate that methylene blue is a safe, low-cost mapping agent with only minimal and transient side effects.

Multivariate logistic regression analysis was performed to explore potential associations between ARM preservation and lymphedema, adjusting for age, BMI, tumor stage, and number of nodes removed. Given the small number of lymphedema events (n = 6), the model is exploratory and may be unstable due to limited events-per-variable, and results should be interpreted cautiously. ARM preservation was associated with lower odds of developing lymphedema (adjusted OR = 0.21, 95% CI: 0.05-0.88, Wald χ² = 4.59, p = 0.032), while none of the other variables showed statistically significant associations: age >50 years (χ² = 0.04, p = 0.85), BMI >30 kg/m² (χ² = 0.38, p = 0.54), Stage III disease (χ² = 0.68, p = 0.41), and >15 nodes removed (χ² = 0.97, p = 0.33). These results should be interpreted as preliminary trends rather than definitive causal effects.

**Table 3:** Exploratory multivariate logistic regression for predictors of lymphedema (n = 60) Odds ratios are presented relative to the specified reference categories. Due to the limited number of lymphedema events (n = 6), this regression analysis is exploratory and at high risk of overfitting. Associations should be interpreted as preliminary trends rather than definitive causal relationships.

Variable	Reference Category	Adjusted OR	95% CI	Statistical Test	Test Value	p-value
ARM Preserved	ARM Sacrificed/Not Identified	0.21	0.05 – 0.88	Wald Chi-square	χ² = 4.59	0.032
Age >50 years	Age ≤50 years	1.12	0.35 – 3.57	Wald Chi-square	χ² = 0.04	0.85
BMI >30 kg/m²	BMI ≤30 kg/m²	1.48	0.41 – 5.28	Wald Chi-square	χ² = 0.38	0.54
Tumor Stage III	Stage I–II	1.66	0.48 – 5.71	Wald Chi-square	χ² = 0.68	0.41
>15 Nodes Removed	≤15 Nodes Removed	1.83	0.52 – 6.44	Wald Chi-square	χ² = 0.97	0.33
Adjuvant Chemotherapy Received	No chemotherapy	1.29	0.36 – 4.61	Wald Chi-square	χ² = 0.21	0.65
Regional Nodal Radiotherapy	No radiotherapy	2.05	0.55 – 7.63	Wald Chi-square	χ² = 1.02	0.31
Postoperative Physiotherapy	No physiotherapy	0.91	0.23 – 3.57	Wald Chi-square	χ² = 0.02	0.88

## Discussion

In this study, ARM using methylene blue dye was successfully performed in the majority of patients, with a preservation rate of 90.2% among identified lymphatics. ARM preservation significantly reduced the incidence of post-mastectomy lymphedema (4.3% vs. 28.6% in the sacrificed/not identified group, p = 0.012) and limited the progressive increase in arm circumference over six months. Multivariate analysis confirmed that ARM preservation independently decreased the odds of developing lymphedema (adjusted OR = 0.21, 95% CI: 0.05-0.88, p = 0.032), while other factors such as age, BMI, tumor stage, and number of lymph nodes removed were not significant predictors. Additionally, no major adverse events were associated with methylene blue, indicating its safety as a low-cost alternative for lymphatic mapping. Overall, these results suggest that methylene blue-based ARM is a practical, safe, and efficient approach to reduce post-operative lymphedema in resource-constrained settings.

The identification and preservation rates in this study are comparable to those reported in previous ARM studies using either radiotracers or dye-based techniques, confirming high feasibility and reproducibility [15]. It is important to note that the extent of damage to lymphedema is considerably reduced compared to the literature, where preservation of ARM lymphatics during axillary removal safeguards lymphatic drainage and reduces arm edema [16]. Serial arm circumference measurement also supports the fact that ARM maintenance averts progressive lymphedema in the short term [17].

The evidence of safety outcomes, such as a lack of systemic toxicity or severe allergic reaction, confirms the use of methylene blue as an alternative to radiotracers, which is convenient and well-tolerated [18]. Moreover, the result of minimal incidence of mild transient discoloration and injection-site discomfort supports prior findings that dye-based mapping is minimally invasive and acceptable to patients [19]. Recent studies also suggest that ARM is safe when used alongside sentinel lymph node biopsy without increasing axillary recurrence rates, enhancing its oncological safety [20]. The overall effect of these findings supports the use of ARM to prevent post-mastectomy lymphedema while maintaining oncological safety.

Despite these positive findings, several limitations warrant discussion. First, the study was a single-arm, prospective observational design, and the allocation into ARM-preserved versus ARM-sacrificed/not identified categories was determined intraoperatively based on lymphatic proximity to tumor-bearing nodes and predefined oncologic criteria. This selection bias may have influenced the risk of lymphedema independently of ARM preservation, as patients in the ARM-sacrificed group may have undergone more extensive axillary dissection [10,11,15]. Second, although multivariate logistic regression was performed to adjust for potential confounders, the analysis included only six total lymphedema events. Therefore, the regression is exploratory and underpowered, and results should be interpreted as preliminary associations rather than definitive causal relationships [14,15]. Other unmeasured predictors, such as regional nodal irradiation, number of positive lymph nodes, adjuvant chemotherapy details, and adherence to postoperative physiotherapy, could further influence lymphedema risk and were not systematically collected. Third, lymphedema assessment relied on manual arm circumference measurements at predefined landmarks performed by a clinician who was not blinded to ARM status. This introduces the potential for measurement and observer bias, particularly since the differences in arm circumference between groups were relatively small (e.g., 0.4 vs. 0.9 cm). Future studies should consider standardized, blinded, or repeated measurements to enhance reliability and reproducibility. Fourth, the follow-up duration was limited to six months. While early postoperative trends are encouraging, late-onset lymphedema may develop months or years after surgery. Claims regarding “long-term” prevention should therefore be interpreted cautiously and are primarily inferred from prior literature rather than this cohort [7,11,18,19]. Fifth, while no major adverse events were observed, rare risks of methylene blue, such as allergic reactions, skin necrosis, or serotonin syndrome in patients on serotonergic medications, should be acknowledged. Inclusion of patient-reported outcomes, including quality-of-life and functional assessments, would provide a more comprehensive evaluation of the clinical impact of ARM preservation, which was not captured in this study.

Future research should focus on multicenter trials with larger cohorts, longer follow-up periods, standardized and blinded outcome assessments, and incorporation of functional and quality-of-life measures. Additionally, combining ARM with advanced lymphatic imaging techniques or evaluating alternative dye concentrations may further optimize identification and preservation rates. Collectively, these findings support the use of methylene blue-based ARM to reduce early post-mastectomy lymphedema while maintaining oncological safety in resource-limited settings [10,11,14,15,18-20].

## Conclusions

The application of ARM using methylene blue dye is a safe and feasible method to identify and preserve arm lymphatics during axillary lymph node dissection. Preservation of ARM lymphatics is associated with a lower incidence of early post-mastectomy lymphedema and reduced progressive arm swelling, without compromising oncologic safety. While methylene blue-based ARM may offer practical advantages in resource-limited settings, claims regarding cost-effectiveness should be interpreted cautiously, as no formal economic analysis was performed in this study. The single-center design and the surgical team’s experience with ARM may limit generalizability, particularly to centers with less expertise or variable intraoperative support. Finally, although improved postoperative outcomes are suggested, quality-of-life benefits were not assessed in this study and should be evaluated in future research. Overall, methylene blue-based ARM represents a promising approach to support lymphatic preservation in breast cancer surgery, warranting further validation in multicenter trials with broader patient populations.
